# Daily survey participation and positive changes in mental health symptom scores among Royal Canadian Mounted Police Cadets

**DOI:** 10.3389/fpsyg.2023.1145194

**Published:** 2023-08-04

**Authors:** Robyn E. Shields, Taylor A. Teckchandani, Gordon J. G. Asmundson, Jolan Nisbet, Rachel L. Krakauer, Katie L. Andrews, Kirby Q. Maguire, Laleh Jamshidi, Tracie O. Afifi, Lisa M. Lix, Alain Brunet, Shannon Sauer-Zavala, Gregory P. Krätzig, J. Patrick Neary, Jitender Sareen, R. Nicholas Carleton

**Affiliations:** ^1^Canadian Institute for Public Safety Research and Treatment (CIPSRT), University of Regina, Regina, SK, Canada; ^2^Anxiety and Illness Behaviours Lab, Department of Psychology, University of Regina, Regina, SK, Canada; ^3^Department of Community Health Sciences, University of Manitoba, Winnipeg, MB, Canada; ^4^McGill’s Psychiatry Department and Douglas Institute Research Center, Montréal, QC, Canada; ^5^Department of Psychology, University of Kentucky, Lexington, KY, United States; ^6^Department of Psychology, University of Regina, Regina, SK, Canada; ^7^Faculty of Kinesiology and Health Studies, University of Regina, Regina, SK, Canada; ^8^Department of Psychiatry, Department of Community Health Sciences, University of Manitoba, Winnipeg, MB, Canada

**Keywords:** mental health monitoring, self-report, mental health disorder symptoms, Royal Canadian Mounted Police, cadets

## Abstract

**Introduction:**

Royal Canadian Mounted Police (RCMP) officers self-report high levels of mental health disorder symptoms, such as alcohol use disorder, generalized anxiety disorder, major depressive disorder, panic disorder, and posttraumatic stress disorder. Participation in regular mental health monitoring has been associated with improved mental health disorder symptom reporting and may provide an accessible tool to support RCMP mental health. The current study assessed relationships between self-reported mental health disorder symptoms and the completion of daily surveys (i.e., daily mental health disorder symptom monitoring) by RCMP cadets during the Cadet Training Program (CTP).

**Methods:**

Participants were RCMP cadets (*n* = 394; 76.1% men) in the Standard Training Program who completed the 26-week CTP and daily self-monitoring surveys, as well as full mental health assessments at pre-training (i.e., starting the CTP) and pre-deployment (i.e., ~2 weeks prior to deployment to the field). Symptoms of alcohol use disorder, generalized anxiety disorder, major depressive disorder, panic disorder, and posttraumatic stress disorder were assessed. Changes in mental health disorder symptom reporting from pre-training to pre-deployment were calculated. Spearman’s rank correlations were estimated for number of daily surveys completed and change in mental health disorder symptom scores between pre-training and pre-deployment.

**Results:**

There were statistically significant inverse relationships between number of daily surveys completed and number of mental health disorder symptoms reported; specifically, cadets who completed more daily surveys during CTP reported fewer symptoms of alcohol use disorder, generalized anxiety disorder, major depressive disorder, panic disorder, and posttraumatic stress disorder.

**Conclusion:**

An inverse correlation between number of daily surveys completed and mental health disorder symptom scores indicated that participation in daily mental health monitoring was associated with improvements in self-reported mental health disorder symptoms between pre-training and pre-deployment. Regular self-monitoring of mental health disorder symptoms may help to mitigate mental health challenges among RCMP cadets and officers.

## 1. Introduction

Public safety personnel [PSP; e.g., correctional workers, firefighters, police officers, paramedics, Royal Canadian Mounted Police (RCMP)] work to protect the safety and security of Canadians (Canadian Institute for Public Safety Research and Treatment, 2020). As a result of their occupational duties, PSP are regularly exposed to potentially psychologically traumatic events (PPTE). PPTE include, but are not limited to, actual or threatened death, serious injury, sexual violence, and military combat (APA, 2022). Frequent exposures to PPTE are associated with an increased risk for symptoms of mental health disorders, collectively referred to in Canada as posttraumatic stress injuries (PTSI; Canadian Institute for Public Safety Research and Treatment, 2020). PTSIs, such as alcohol use disorder (AUD), generalized anxiety disorder (GAD), major depressive disorder (MDD), panic disorder (PD), and posttraumatic stress disorder (PTSD) are more prevalent in PSP than the general population (Carleton et al., 2018), with 44.5% of PSP in Canada screening positively for one or more PTSI. RCMP officers screen positively (50.2%) more frequently than the average for PSP occupational groups (Carleton et al., 2018).

The high prevalence of positive screens for PTSI among active duty RCMP members is likely due, in part, to frequent and diverse PPTE exposures. More than 95% of RCMP officers report at least one PPTE exposure, though, on average, they endorse exposure to more than 11 different types of PPTE (Carleton et al., 2019). Indeed, more than half of RCMP officers report exposure to sudden accidental death (59.7%), sudden violent death (56.5%), physical assault (54.9%), and serious transportation accident (73.5%; Carleton et al., 2019). Accordingly, many active duty RCMP members report symptoms of mental health disorders, such as AUD (3.9%), GAD (23.3%), MDD (31.7%), PD (12%), and PTSD (30%; Carleton et al., 2018). RCMP cadets also self-report mental health disorder symptoms (Carleton et al., in press) and PPTE exposure histories (Andrews et al., 2023), but less frequently than serving RCMP, likely due to less occupational experience. Given the occupational reality of frequent PPTE exposures for RCMP, identifying accessible tools to mitigate PTSI is imperative for protecting their mental health.

Self-monitoring of mental health disorder symptoms has been associated with decreasing such symptoms (Kauer et al., 2012). Health-focused self-monitoring in areas such as weight loss (Burke et al., 2011), alcohol use (Kavanagh et al., 1999), and sleep hygiene (Todd and Mullan, 2014) improves self-reflection and encourages healthy habits. Self-monitoring of mood is a common technique used as a part of different therapy modalities, including cognitive behavioral therapy (CBT; Cohen et al., 2013), dialectical behavioral therapy (DBT; Feldman et al., 2009), and acceptance and commitment therapy (ACT; Hayes et al., 2012). Clients who keep track of their thoughts, feelings, and behaviors are more likely to actively notice and acknowledge their emotions, thereby practicing emotional self-awareness (Cohen et al., 2013). Increased emotional awareness and knowledge about one’s symptoms can facilitate restructuring of maladaptive anxiety responses, challenging of depression perpetuating behaviors (Jarrett and Nelson, 1987), and treating PTSD (Tarrier et al., 1999; Ehlers et al., 2003). Accordingly, daily mental health disorder symptom monitoring can take as little as 60 s and can be a quick and easy way for RCMP cadets and officers to monitor their mental health and encourage proactive help-seeking if needed.

As a part of the larger RCMP Study (Carleton et al., 2022), participants were invited to complete brief daily self-assessments to monitor their physical and mental health, and reflect on the associated changes over time. Participants were encouraged to consider and record their mood, physical wellness, emotional state, sleep hours, sleep quality, physical activity, and substance use (i.e., alcohol, tobacco, illicit substances). The current study was designed to examine the relationship between the frequency of daily mental health monitoring and changes in mental health disorder symptom reporting of participating cadets between pre-training [i.e., the start of the Cadet Training Program (CTP)] and pre-deployment (i.e., ~24 weeks later, which is ~2 weeks prior to deployment to the field). Based on the extant literature (e.g., Ehlers et al., 2003; Kauer et al., 2012; Murnane et al., 2016; Eisenstadt et al., 2021; Gatto et al., 2022), cadets who completed more daily surveys were expected to report fewer mental health disorder symptoms.

## 2. Materials and methods

### 2.1. Procedure

The current study is a part of the longitudinal RCMP Study, with full procedural details available in a published protocol paper (Carleton et al., 2022). The RCMP Study was approved by the University of Regina Institutional Research Ethics Board (file No. 2019–055) and the RCMP Research Ethics Board (file No. SKM_C30818021312580). The RCMP Study was also approved through a Privacy Impact Assessment as part of the overall approval including the National Administrative Records Management System (NARMS) file No. 201611123286 and Public Services and Procurement Canada (PSPC) file No. 201701491/M7594174191. Mental health disorder symptom measures and daily survey data were collected via online surveys. Individual participants were provided with automated feedback through a secured web portal.

### 2.2. Sample and data

Participants for the current study were a sub-sample of RCMP Study cadet participants (*n* = 394) who completed the 26-week CTP as part of the Standard Training Program and completed at least one daily survey during the CTP, as well as Full Surveys at pre-training (i.e., starting the CTP) and pre-deployment (i.e., ~2 weeks prior to deployment to the field). Cadets were Canadian citizens or permanent residents, 19 to 57 years old, who can fluently read, write, and speak either English or French (Hembroff and Krätzig, 2020). Cadets must meet several recruiting requirements, including security clearances, medical examinations, a polygraph test, and minimum physical standards. There were no other conditions requiring exclusion of cadets otherwise qualified for the CTP and participating in the RCMP Study as part of the Standard Training Program (Carleton et al., 2022).

### 2.3. Self-report measures

#### 2.3.1. Daily Surveys

The Daily Surveys were created for the RCMP Study (Carleton et al., 2022). Daily Surveys are brief self-report questionnaires that ask cadets to report on the previous 24-h period, recording scores in the domains of mood, attitude, performance, physical wellness, emotional state, hours worked, hours slept, quality of sleep, eating patterns, social activity, physical activity, and substance use. Details on response options for each area queried can be found in the published protocol paper (Carleton et al., 2022). The current work assesses associations with self-monitoring as a function of completing the Daily Surveys (i.e., participation), rather than associations with the specific content of the Daily Survey responses. The Daily Surveys are a new measure; specific psychometric details and analyses will be available as soon as possible in a dedicated psychometric paper. In the interim, further details can be found in the associated protocol paper (Carleton et al., 2022). Daily Surveys take approximately 1 min to complete via smart phone.

#### 2.3.2. Alcohol Use Disorder Identification Test

The Alcohol Use Disorder Identification Test (AUDIT; Saunders et al., 1993) is a 10-item self-report questionnaire assessing alcohol intake, alcohol dependence, and adverse consequences of alcohol use over the past 12 months. Respondents rate items such as “How many drinks containing alcohol do you have on a typical day?” on a 5-point Likert-like scale (i.e., 0 = *never* to 4 = *daily or almost daily*). Psychometric evaluation of the AUDIT has demonstrated good internal consistency (α = 0.85) in the general population (Daeppen et al., 2000; Reinert and Allen, 2007) and in police populations (α = 0.81; Davey et al., 2000).

#### 2.3.3. Generalized Anxiety Disorder Scale-7

The Generalized Anxiety Disorder Scale-7 (GAD-7; Spitzer et al., 2006) is a 7-item self-report measure assessing symptoms of anxiety and worry. Participants are asked to rate their experiences of symptoms over the last 2 weeks (e.g., “Not being able to stop or control worrying”) on a Likert-like scale (i.e., 0 = *not at all* to 3 = *nearly every day*). The GAD-7 has demonstrated good internal consistency (α = 0.89) in a community sample (Löwe et al., 2008) and in a police sample (α = 0.93; Korol et al., 2021).

#### 2.3.4. Panic Disorder Severity Scale-Self-Report

The Panic Disorder Severity Scale-Self-Report (PDSS-SR; Shear et al., 1997) is a 7-item self-report measure designed to assess symptoms of panic disorder. Respondents rate items (e.g., “How many panic and limited symptom attacks did you have during the past 7 days?”) on a 5-point Likert scale (i.e., 0 = *none* to 4 = *extreme*). The self-report version of the PDSS-SR has displayed excellent internal validity in a clinical population (α = 0.92; Houck et al., 2002) and with a police population (α = 0.93; Korol et al., 2021).

#### 2.3.5. Patient Health Questionnaire-9

The Patient Health Questionnaire-9 (PHQ-9; Kroenke et al., 2001) is a 9-item self-report questionnaire that assesses symptoms of MDD. Items (e.g., “Little interest or pleasure in doing things”) are rated on a 4-point Likert scale (i.e., 0 = *not at all* to 3 = *nearly every day*). Psychometric evaluation found the PHQ-9 to be a valid measure of depression symptoms and severity, with good internal consistency (α = 0.89) in the general population (Kroenke et al., 2001) and police populations (α = 0.91; Korol et al., 2021).

#### 2.3.6. PTSD Checklist for DSM-5

The PTSD Checklist for DSM-5 (PCL-5; Blevins et al., 2015) is a 20-item self-report measure used to assess symptoms of PTSD. Participants use a Likert scale (i.e., 0 = *not at all* to 4 = *extremely*) to rate how bothered they had been by PTSD symptoms (e.g., “Repeated, disturbing dreams of the stressful experience”) over the past month. Psychometric evaluation has found the PCL-5 to be a reliable and valid measure of PTSD, with strong internal consistency (α = 0.94) in PPTE-exposed populations (Blevins et al., 2015).

### 2.4. Sociodemographic variables

Sociodemographic characteristics, including sex (i.e., male and female), gender (i.e., man, woman), age (i.e., 19 to 29 years, 30 to 39 years, 40 to 49 years, and 50 to 59 years), marital status (i.e., single, separated/divorced, and married/common-law), province of residence [i.e., Western Canada (British Columbia, Alberta, Saskatchewan, Manitoba), Eastern Canada (Ontario, Quebec), Atlantic Canada (Newfoundland & Labrador, Prince Edward Island, Nova Scotia, New Brunswick) or Northern Territories (Yukon, Northwest Territories, Nunavut)], and highest level of education completed (i.e., high school graduate or less, some post-secondary school, and university degree/4-year college or higher), were used to characterize the current study sample.

### 2.5. Statistical analyses

Participant sociodemographic variables were described using frequencies and percentages. Mean, standard deviation, skew, and kurtosis for the number of daily surveys completed for the total sample were calculated. A series of *t*-tests and analysis of variance (ANOVA) tests were used to assess differences in symptoms reported and daily survey completion across sociodemographic groups. Cohen’s *d* statistics, standardized effect sizes, were calculated for two-group differences and $\eta_{p}^{2}$ statistics, standardized effect sizes, were calculated for multi-group differences (Cohen, 1988). Cohen (1988) guidelines for small (*d* = 0.2), medium (*d* = 0.5), and large (*d* = 0.8) effect sizes were used. Holm-Bonferroni adjustments were applied to control for familywise error rate in multiple comparisons from *post hoc* testing. Changes in mental health disorder symptom scores (i.e., AUDIT, GAD-7, PHQ-9, PDSS-SR, and PCL-5) between the start of CTP and pre-deployment were calculated by subtracting pre-training scores from pre-deployment scores. The distribution of the data was better suited to a Spearman’s Rank correlation due to the monotonic relationship rather than the assumption of linearity; therefore, Spearman’s rank correlations were estimated between number of daily surveys completed and changes in self-reported mental health disorder symptom scores based on the AUDIT, GAD-7, PHQ-9, PDSS-SR, and PCL-5.

## 3. Results

Table 1 includes details of self-reported participant sociodemographic characteristics and symptom scores. Participants were mostly men (76.1%), between the ages of 19 to 29 years (56.3%), and married/common-law (50.5%) or single (47.7%). No participants reported being widowed. Biological sex and gender were both queried at pre-training. All participants who identified as male also identified as a man and all participants who identified as female also identified as a woman; therefore, only gender is reported in Table 1. No participants identified with a non-binary gender. Participants were mainly from Western Canada (77.9%; i.e., British Columbia, Alberta, Saskatchewan, Manitoba) and reported having either completed some post-secondary school (48.5%) or a university degree, 4-year college, or higher level of education (44.6%). Most participants (58.1%) did not have prior PSP experience.

**Table 1:** Sociodemographic characteristics and comparisons of mental health disorder symptom measure change scores.

	% (*n*)^1^	Number of daily surveys completed	AUDIT	GAD-7	PHQ-9	PDSS-SR^2^	PCL-5
		*Mdn* (IQR)	*M* (*SD*)	*M* (*SD*)	*M* (*SD*)	*M* (*SD*)	*M* (*SD*)
Mean mental health disorder symptom total scores							
**Total sample**							
Pre-training	100 (394)		2.83 (2.61)	4.02 (4.09)	3.01 (3.42)	0.39 (1.71)	5.34 (9.05)
Pre-deployment	100 (394)		0.55 (1.48)	0.69 (2.03)	0.60 (1.82)	0.01 (0.20)	0.67 (3.40)
Comparisons of mental health disorder symptom change scores							
**Total sample**							
	100 (394)	24.00 (44.00)	−0.27 (1.97)	−1.03 (3.73)	−0.54 (3.29)	−2.80 (5.63)	−2.13 (7.67)
**Gender**							
Men	76.1 (300)	23.00 (49.00)	−2.21 (2.54)	−3.22 (4.00)	−2.41 (3.31)	−0.18 (1.10)	−4.23 (8.27)
Women	23.9 (94)	24.00 (39.75)	−2.34 (2.69)	−3.51 (4.82)	−2.26 (4.43)	−0.76 (2.28)	−6.03 (12.32)
Effect Size (Cohen’s *d*)		0.002	0.001	0.001	0.000	0.017*	0.006
**Age group (years)**							
19–29	56.3 (222)	25.00 (45.00)	−2.40 (2.71)	−3.17 (4.24)	−2.34 (3.87)	−0.31 (1.45)	−4.46 (9.31)
30–39	35.0 (138)	22.00 (45.00)	−1.89 (2.34)	−3.64 (4.32)	−2.57 (3.41)	−0.42 (1.74)	−5.19 (9.99)
40–49	7.9 (31)	22.00 (48.50)	−2.54 (2.26)	−3.00 (3.57)	−1.67 (1.76)	0	−4.62 (8.71)
50–59	^	49.50 (55.00)	−2.50 (3.53)	−1.00 (1.41)	−1.50 (0.71)	0	0
Effect size ($\eta_{p}^{2}$)		0.001	0.008	0.004	0.003	0.003	0.002
**Marital status**							
Single	47.7 (188)	21.50 (42.25)	−2.46 (2.85)	−3.44 (4.01)	−2.57 (3.96)	−0.40 (1.56)	−5.53 (10.24)
Separated/divorced	1.8 (7)	10.00 (31.75)	−3.00 (3.58)	−6.37 (4.8)	−2.87 (2.17)	-	−7.37 (9.30)
Married/Common-Law	50.5 (199)	28.00 (54.00)	−2.01 (2.21)	−3.02 (4.34)	−2.14 (3.29)	−0.26 (1.48)	−3.73 (8.59)
Effect size ($\eta_{p}^{2}$)		0.005	0.009	0.015	0.006	0.004	0.013
**Province of residence**							
Western Canada (BC, AB, SK, MB)	77.9 (307)	27.50 (43.75)	−2.24 (2.46)	−3.67 (4.53)	−2.69 (3.64)	−0.42 (1.72)	−5.47 (10.24)
Eastern Canada (ON, QC)	16.5 (65)	18.50 (40.00)	−2.18 (2.71)	−2.78 (3.95)	−1.89 (3.83)	−0.21 (1.05)	−3.34 (8.57)
Atlantic Canada (PEI, NS, NB, NFL)	5.1 (20)	32.00 (59.50)	−2.50 (2.88)	−3.39 (3.44)	−2.52 (2.67)	−0.26 (1.75)	−5.00 (7.62)
Northern Territories (YK, NWT, NVT)	^	11.00 (23.00)	−2.00 (1.23)	−2.69 (1.51)	−1.00 (0.70)	0	−6.20 (11.15)
Effect size ($\eta_{p}^{2}$)		0.002	0.003	0.012	0.009	0.001	0.009
**Education**							
High school graduate or less	6.9 (27)	16.00 (50.00)	−2.20 (2.25)	−3.62 (4.31)	−2.68 (2.91)	−0.64 (2.11)	−4.62 (8.81)
Some post-secondary school	48.5 (191)	24.00 (44.00)	−2.45 (2.88)	−3.74 (4.38)	−2.61 (3.70)	−0.11 (1.08)	−5.20 (9.32)
University degree/4-year college or higher	44.6 (176)	26.00 (45.00)	−2.04 (2.32)	−2.65 (3.94)	−1.97 (3.72)	−0.42 (1.59)	−3.73 (9.39)
Effect size ($\eta_{p}^{2}$)		0.001	0.004	0.014	0.008	0.010	0.010
**Prior PSP service**							
Yes	31.9 (126)	24.00 (43.50)	−2.16 (3.18)	−2.76 (4.48)	−2.38 (3.76)	−0.31 (1.55)	−5.11 (10.33)
No	58.1 (229)	21.00 (49.00)	−2.37 (2.21)	−3.49 (4.03)	−2.34 (3.57)	−0.36 (1.57)	−4.36 (9.12)
Effect size (Cohen’s *d*)		0.001	0.008	0.012	0.007	0.002	0.002

AUDIT, Alcohol Use Disorder Identification Test; GAD-7, Generalized Anxiety Disorder Scale-7; *M* (*SD*), mean (standard deviation); $\eta_{p}^{2}$, partial Eta squared; PDSS-SR, Panic Disorder Symptoms Scale - Self-Report; PHQ-9, Patient Health Questionnaire-9; PCL-5, Posttraumatic Stress Disorder Checklist for DSM-5.

1 Total percentages may not sum to 100 and ns may not sum to 394 due to non-response or responding “other.”

2 A limited number of participants reported values for panic disorder (PDSS-SR) because selecting “No” for “ever having experience with panic attacks” or “having a panic attack in the last 7 days,” meant participants were not presented the rest of the PDSS-SR questions.

^ *n* < 5, data not presented.

* *p* ≤ 0.01.

There were no statistically significant differences between men and women in the AUDIT, GAD-7, PHQ-9, or PCL-5 change scores. Women reported statistically significantly greater change scores on the PDSS-SR (*p* < 0.01), but the effect size was small (*d* = 0.02). There were no statistically significant differences across age group, marital status, province of residence, or education level with respect to self-report symptom measure change scores (all *p*s > 0.05). There were also no statistically significant differences across all sociodemographic characteristics with respect to number of daily surveys completed (all *p*s > 0.05).

A total of 15,400 daily surveys were completed by cadets, with a median of 24 surveys and an interquartile range of 44 surveys. The data were positively skewed, 2.35(0.12), with heavy tails, 9.63(0.25). Results evidenced statistically significant inverse relationships between self-reported mental health disorder symptom change scores and daily survey completion, such that cadets who completed more daily surveys during CTP reported decreases in all measured self-reported mental health disorder symptom scores (*p*s < 0.01). Cadets who completed more daily surveys during CTP had greater decreases in their AUDIT scores (ρ = −0.196, *p* < 0.001), GAD-7 scores (ρ = −0.522, *p* < 0.001), PHQ-9 scores (ρ = −0.488, *p* < 0.001), PDSS-SR scores (ρ = −0.108, *p* < 0.01), and PCL-5 scores (ρ = −0.383, *p* < 0.001).

## 4. Discussion

The current study results indicated that RCMP cadets who completed more daily surveys self-monitoring their mental health also had greater decreases in their self-reported symptoms of several mental health disorders (i.e., AUD, MDD, GAD, PD, and PTSD) from pre-training (i.e., starting the CTP) to pre-deployment (i.e., ~2 weeks prior to deployment to the field). The absolute causality of the relationship in the current study is unknowable without a randomized controlled trial. Participants with fewer mental health disorder symptoms may participate more in self-monitoring, leading to increased self-awareness and decreased mental health disorder symptoms. The differences in mental health disorder symptoms scores from pre-training to pre-deployment were not clinically significant; however, this is to be expected, as participating RCMP cadets report overall low levels of mental health disorder symptoms (Carleton et al., in press). While not clinically significant in RCMP cadets, the relationship between daily mental health monitoring and positive changes in mental health disorder symptoms provides a potential avenue for active duty RCMP officers to mitigate high levels of mental health disorder symptoms. As mental health disorder symptoms occur along a spectrum, mechanisms for change at one end of the spectrum (i.e., lower levels of mental health disorder symptoms) can reasonably be assumed to be mechanisms for change at the other end of the spectrum (i.e., higher levels of mental health disorder symptoms).

Participants in the study were instructed to complete one daily survey per day; however, surveys were not completed with regularity during training. Instead, participants tended to complete more daily surveys at certain time points throughout their training, speculatively at times when training demands were lower. To our knowledge, extant literature does not provide a minimum threshold for daily survey completion that would produce positive changes in mental health disorder symptoms. Identifying a threshold number of daily surveys needed to see improvements in mental health disorder symptoms is outside the scope of the current study; however, the inverse association between daily survey participation and number of self-reported mental health disorder symptoms is consistent with the pre-registered hypotheses for the RCMP Study (Carleton et al., 2022) and with previous research that suggests self-monitoring mental health disorder symptoms may help improve mental health (Ehlers et al., 2003; Kauer et al., 2012; Murnane et al., 2016; Eisenstadt et al., 2021; Gatto et al., 2022). A nuanced temporal analysis of survey completion was also outside the scope of the current study but is available in a related study (Teckchandani et al., n.d.).

There were no differences across sociodemographic groups (i.e., gender, age group, marital status, province of residence, education level, or prior PSP service) with respect to changes in mental health disorder symptom scores, except for changes in scores on the PDSS-SR. Women had greater improvements in their PD symptoms than did men; however, the effect size was small and there may have been a floor effect, as very few RCMP cadets report symptoms of PD (Carleton et al., in press). There were also no differences across sociodemographic characteristics with respect to number of daily surveys completed. Although not statistically significant, women in the current study completed more daily surveys than did men. Research on regular mental health monitoring has frequently reported biased samples (i.e., mostly women) and as such, gender comparisons have often been left out (Eisenstadt et al., 2021). Women PSP appear more willing to report mental health disorder symptoms than men (Carleton et al., 2018; Krakauer et al., 2020), which may be associated with being more willing to complete regular mental health monitoring. Regardless of sociodemographic differences, all participants in the current study who engaged in daily monitoring of their mental health disorder symptoms had improvements in such symptoms, suggesting that the improvements seen in mental health disorder symptoms from daily monitoring are beneficial to all RCMP cadets who engage in daily monitoring.

### 4.1. Strengths and limitations

The overall RCMP Study has several strengths, which can be found in the published protocol paper (Carleton et al., 2022). Specific to the current study, strengths included: 1) cadets were given internet capable devices with which to complete daily surveys, removing any financial barriers to participation and 2) the collection of baseline data allows researchers to monitor participation and identify changes in participation patterns that could signal early development of PTSIs.

Limitations of the overall RCMP Study can be found in the published protocol paper (Carleton et al., 2022). Limitations specific to the current study include: 1) voluntary participation in the study and for completing the daily self-monitoring surveys, leading to self-selection biases potentiating unknowable influences (e.g., participants with better mental health may have self-selected into the study and been better able to complete the surveys); 2) participants were not provided with a daily reminder to complete their daily survey, which may have limited the results and created an unknowable bias based on volitional participation (e.g., participants with better mental health may have been better able to remember to participate); 3) the sample was mostly men, limiting the ability to do gender comparisons or stratification; 4) data on mental health treatment were not collected; therefore, there is no way of knowing whether or not the cadets sought treatment for their mental health and, in turn, experienced decreases in reported symptoms as a result; 5) floor effects limited the detectability of clinically significant changes in mental health disorder symptoms scores; and 6) attritional data were not collected; therefore, participants with more mental health disorder symptoms may have left the study, which may have contributed to the floor effects.

### 4.2. Future directions

Future researchers should analyze attrition throughout CTP and RCMP officers’ careers by using survival analysis based on mental health disorder symptoms measures. The effects of underlying mental health disorder symptoms and availability of mental health supports on an RCMP officer’s disposition to sustain a mental health injury in the line of duty can be learned from analyses comparing how cadets who screen positively for mental health disorder symptoms attrition from CTP or the RCMP Study. Multifactorial models could be used to explore the latent variables underpinning the relationships between participation in self-monitoring and self-report mental health disorder symptom changes during CTP. Further investigations could assess the moderating effects of variables such as social support (Takagi et al., 2013), suicidal ideation (Hoge et al., 2002), mental health history (Hoge et al., 2002), and engagement in physical activity (Garcia et al., 2015), informing targeted mental health supports for RCMP cadets and officers.

## 5. Conclusion

The data indicated an inverse relationship between mental health symptom scores and the number of daily surveys completed by cadets during CTP, when comparing cadet scores reported pre-training (i.e., starting the CTP) and pre-deployment (i.e., ~2 weeks prior to deployment to the field). The results were consistent with extant evidence that daily self-monitoring of mental health disorder symptom can improve mental health disorder symptoms through increased emotional awareness (Kauer et al., 2012), knowledge of mood patterns, and self-management of mental health disorder symptoms (Caldeira et al., 2017). Due to low self-reported mental health disorder symptoms in RCMP cadets, clinically significant changes in mental health disorder symptoms scores were not seen in the current study, likely due to statistical floor effects. Mental health disorder symptoms occur along a continuum and mechanisms for change seen at the lower end of the spectrum (i.e., results from the current study) can reasonably be assumed to be mechanisms for change at higher ends of the spectrum. Accordingly, daily mental health disorder symptoms monitoring may help to mitigate high levels of mental health disorder symptoms reported by serving RCMP members. More nuanced group-wise and longitudinal analyses may provide unique insights about the relationships between mental health disorder symptom scores and participation. Additional analyses may critically support the recommendation to include self-reflection as a prophylactic component during the CTP to potentially reduce the impact of PPTE throughout the cadets’ careers.

## Data availability statement

The datasets presented in this article are not readily available because the datasets will be made available only for independent confirmation purposes and only to persons with the necessary ethical and security clearances as defined by the research ethics board at the University of Regina and the contractual obligations with the Royal Canadian Mounted Police. Requests regarding the datasets can be made to nick.carleton@uregina.ca.

## Ethics statement

Data for the current paper were collected as a part of the broader RCMP Study. The associated protocol paper provides full details of the RCMP longitudinal Study (Carleton et al., 2022). The RCMP Study was approved by the University of Regina Ethics Board on April 10, 2019 (File #2019-055), and the RCMP Research Ethics Board followed with approval on April 12, 2019 (File #SKM_C30818021312580). The study was also approved through a Privacy Impact Assessment as part of the overall National Administrative Records Management System approval (201611123286) and Public Services and Procurement Canada approval (201701491/M7594174191). The project is bound by the Privacy Act, R.S., 1985, c. P-21 and the Personal Information Protection and Electronic Documents Act, SC. 2000, c.5 and approved by Public Services and Procurement Canada (PSPC) M7594- 171491/001/SS. The participants provided their electronically recorded informed consent to participate in this study.

## Author contributions

RNC, RS, and TT: conceptualization. RNC, RS, TT, TA, GA, GK, and LL: methodology. RNC, RS, TT, and GK: validation. RNC, RS, and TT: formal analysis. RNC, AB, GK, JPN, and SS-Z: investigation. RNC, AB, GK, and SS-Z: resources. RNC, TT, AB, GK, and JPN: data curation. RS, TT, and RNC: writing—original draft preparation. All authors: substantial contributions consistent with the International Committee of Medical Journal Editors, writing—review and editing, and view and approve the submitted version of the manuscript.

## Funding

The RCMP Study was funded by support from the RCMP, the Government of Canada, and the Ministry of Public Safety and Emergency Preparedness. LL was supported by a Tier I Canada Research Chair in Methods for Electronic Health Data Quality. TA was supported by a Tier I Canada Research Chair in Childhood Adversity and Resilience. The development, analyses, and distribution of the current article was made possible by a generous and much-appreciated grant from the Medavie Foundation.

## Conflict of interest

The authors declare that the research was conducted in the absence of any commercial or financial relationships that could be construed as a potential conflict of interest.

## Publisher’s note

All claims expressed in this article are solely those of the authors and do not necessarily represent those of their affiliated organizations, or those of the publisher, the editors and the reviewers. Any product that may be evaluated in this article, or claim that may be made by its manufacturer, is not guaranteed or endorsed by the publisher.
